# High Incubation Attendance and Nesting Site Constraints of the Sclater's Monal in an Alpine Environment in Southwestern China

**DOI:** 10.1002/ece3.70665

**Published:** 2024-12-04

**Authors:** Nehafta Bibi, Ge Gao, Dan Liang, Xu Luo

**Affiliations:** ^1^ Key Laboratory for Forest Resources Conservation and Utilization in the Southwest Mountains of China, Ministry of Education /College of Forestry Southwest Forestry University Kunming China; ^2^ Baoshan Management Bureau of Gaoligongshan National Nature Reserve Baoshan Yunnan China; ^3^ Princeton School of Public and International Affairs Princeton University Princeton New Jersey USA; ^4^ College of Biological Science and Food Engineering Southwest Forestry University Kunming China

**Keywords:** alpine environment, breeding biology, Gaoligong Mountains, nest reuse, Sclater's monal

## Abstract

Breeding in alpine environments poses significant challenges to birds, requiring specific adaptations for survival. The Sclater's monal ( *Lophophorus sclateri* ), a regionally threatened, typical alpine pheasant species, is restricted to high‐elevation habitat from the East Himalayas to the mountains of west Yunnan, China. Due to its low population density and the difficulty of accessing its habitats, the breeding ecology of this species is understudied. Therefore, we aimed to understand the breeding behavior, nest site use, and life‐history traits that allow this monal species to cope with the alpine environment. During our fieldwork from March to June 2015 and 2016 in the Gaoligong Mountains in western Yunnan, China, we found six cliff nesting sites ranging from 3535 m to 3892 m. Three sites were active, with one being used in both years, and the remaining three were inactive but had been used in prior years. The clutch size was 2.75 ± 0.5 (2 or 3 eggs; *n* = 4 nests at three nesting sites), and all 11 eggs were successfully hatched. The female solely performed incubation, spending 97.2% of its time in incubating with an average duration of 25.69 ± 13.79 h (*n* = 43 bouts across three females) per on‐bout which indicates the female bird invested more time than other pheasants. High incubation attendance by females highlights the importance of increased parental care in ensuring reproductive success. These findings highlight that the Sclater monal exhibits specific breeding behaviors and nesting strategies that reflect adaption to harsh environments. Additionally, our observations of intense male–male interactions and reuse of nesting sites suggest that suitable nesting sites are limited, which could significantly impact population dynamics. Together, these insights are crucial for conserving this regionally threatened pheasant.

## Graphical abstract

The study focused on the breeding ecology of the Sclater's monal in the alpine environment of western Yunnan, China. It found that the species has a low population density and restricted nesting sites, with intense male–male competition observed. Females invest significantly more time in incubation, likely to cope with harsh conditions. These findings highlight the importance of understanding avian reproduction ecology for the conservation of threatened species like the Sclater's monal.

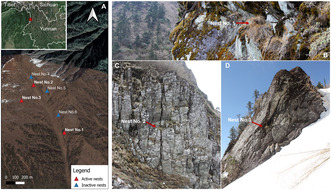

## 1 Introduction

The alpine environment presents significant challenges for breeding birds due to lower temperatures, limited food supplies, and significant climate variations (Martin et al. 2022). These challenging conditions restrict the occurrence of some species and require special adaptations for survival including restricted ranges and unique behaviors to conserve energy and protect their young (Chamberlain et al. 2013). At first glance, rising temperatures might ease these challenges, potentially offering longer breeding seasons and less exposure to freezing conditions. Warming temperatures, however, represent a serious threat to alpine species, whose survival depends on the stability of ice and snow (Hotaling et al. 2017). These elements are crucial for regulating water availability, vegetation growth, and habitat structure, all of which are necessary for maintaining alpine ecosystems. Because warming temperatures or climate change accelerates the melting of snow and ice, the loss of these vital elements leads to habitat degradation, fragmentation, and resource depletion. As a result, alpine species with their narrow ecological niches are more vulnerable to population decline or extinction due to their inability to adjust to warmer conditions (Sandercock, Martin, and Hannon 2005). Thus, climate change, instead of alleviating difficulties, exacerbates the challenges faced by alpine species within the alpine ecosystem.

Due to the difficulty of accessing alpine habitats, there are large gaps in the knowledge of the population status and habitat requirements of alpine breeding species. According to Martin et al. (2022), these gaps obstruct efficient conservation planning and our comprehension of how alpine species adapt to harsh environments. Alpine birds have developed a variety of life history strategies to survive in such extreme environments, balancing their energy requirements with providing sufficient care for their young (Boyle, Conway, and Bronstein 2011). These strategies include building large nests to enhance protection (Liang, Cai, and Yang 2017), laying fewer but larger eggs to improve offspring viability (Badyaev and Ghalambor 2001; Lu et al. 2010), and extending incubation duration followed by feeding chicks for 1 or 2 days (Lu, Ke, and Zeng 2009; Liang, Cai, and Yang 2017; Liang et al. 2020).

In certain species, males and females share equal parenting responsibilities, which can be useful in harsh environments (Gross 2005; Cockburn 2006). In other species, however (Schwagmeyer et al. 1999; Møller and Cuervo 2000), males concentrate on securing and defending their nesting territories, whereas females are responsible for providing parental care. For many species, particularly in alpine environments, this makes the incubation period very challenging, due to the high energy demands.

Certain Galliformes species with uniparental incubation face significant challenges in balancing reproductive efforts with self‐maintenance (Li et al. 2022). To maintain their energy levels, incubating females must carefully balance the trade‐off between foraging (off‐bouts) and nest attendance (on‐bouts). Cold temperatures exacerbate this challenge, as nests lose heat quickly, necessitating shorter foraging trips to prevent egg cooling (Jia, Liu, and Zhang 2010; Jia, Sun, and Swenson 2010; Fu, Dai, et al. 2017; Fu, Liu, et al. 2017). This delicate balance between investing in incubation and accessing resources emphasizes ambient temperature's critical role in influencing these species' incubation behavior (Webb 1987; Deeming 2002; Li et al. 2022). Pheasant species including the Sichuan partridge ( *Arborophila rufipectus* ) and the Blood pheasant ( *Ithaginis cruentus* ) have evolved to the cold by being highly resistant to embryonic hypothermia. Because of this adaptation, females can leave the eggs for foraging for several hours every day (Jia, Liu, and Zhang 2010; Jia, Sun, and Swenson 2010; Fu, Dai, et al. 2017; Fu, Liu, et al. 2017). Hence, despite breeding in similar alpine environments, some Galliformes species have evolved various strategies to cope with challenges such as cold temperatures, food scarcity, and harsh weather. For example, the Chinese Grouse ( *Tetrastes sewerzowi* ) leaves its nests more frequently but takes shorter breaks (Shi et al. 2019); the Snow partridge ( *Lerwa lerwa* ) and the Blood pheasant have developed unique incubation strategies, including bimodal pattern of recess timing and they have lower nest attendance with fewer daily departures (Jia, Liu, and Zhang 2010; Jia, Sun, and Swenson 2010; Li et al. 2022).

Nevertheless, despite these adaptations, little is known about the unique breeding habits and limitations of the Sclater's Monal ( *Lophophorus sclateri* ), a regional threatened species, in alpine environments. To address these gaps, we conducted this study in the middle section of the Gaoligong Mountains from 2015 to 2016. This region has a relatively higher density of Sclater's monal than our previous study site in the southern part of the Gaoligong Mountains (Luo and Han 2011). Our research focused on the breeding ecology of Sclater's monal, specifically examining its nesting habitats. By filling in a significant gap in the literature and advancing our knowledge of how alpine birds adapt to harsh environments, we attempt to explore the high incubation attendance and nest site constraints of the Sclater's Monal in an alpine environment in Southwestern China.

## 2 Methods

### 2.1 Study Site

**FIGURE 1:**
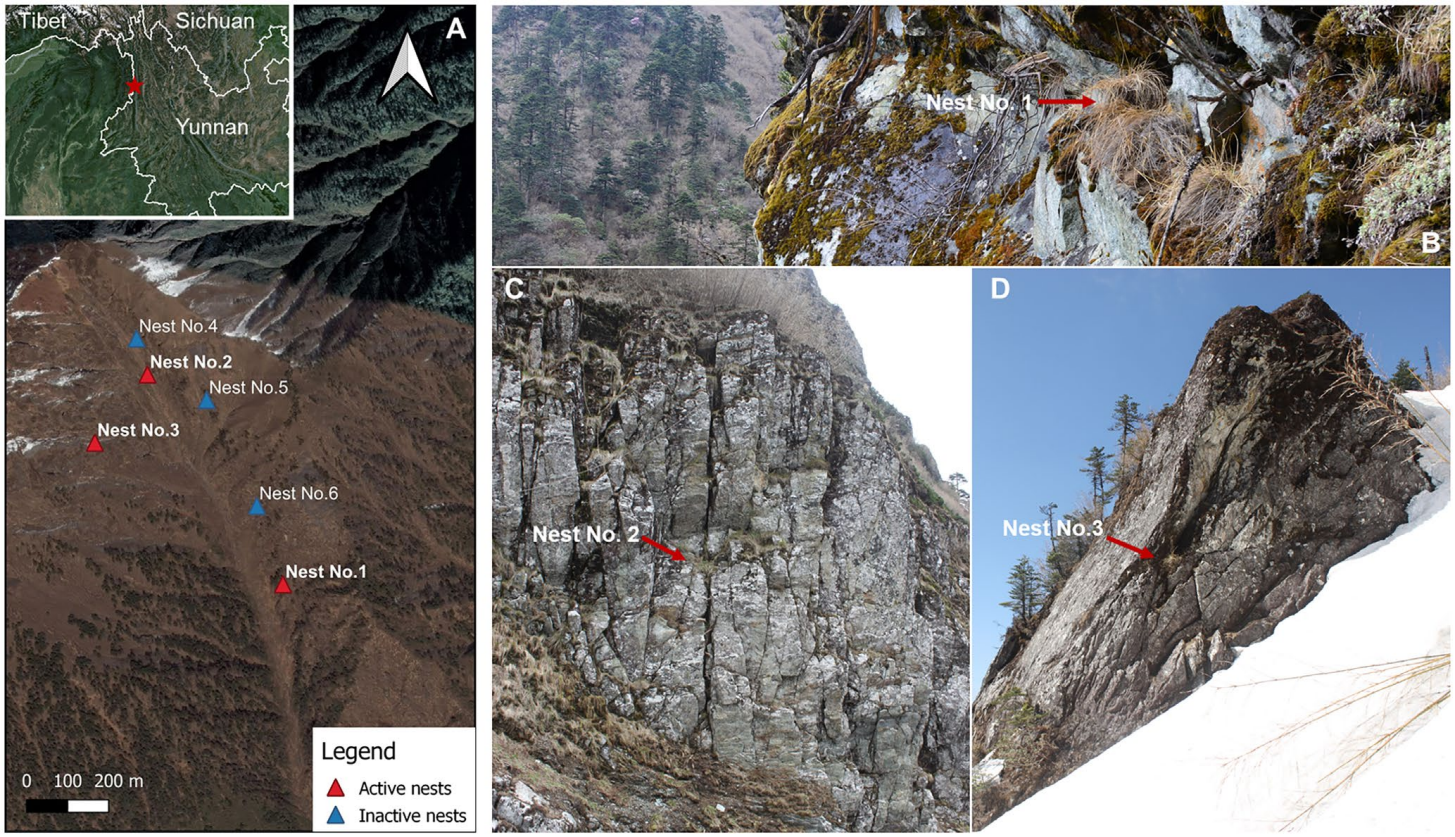
Left panel: Study site and nesting sites. Right panel: Nesting habitat of the Sclater's Monal at a total area of ~5 km^2^.

Our research site is located at an elevation of 3000–4100 m a.s.l. (N26°25′18.2″ E98°47′6.6″), mostly above the tree line, on the eastern slope of the middle section of the Gaoligong Mountains in southwest China (Figure 1). It is situated in the core zone of the Gaoligongshan National Nature Reserve (Ai 2006). We selected this study site based on prior conversations with reserve staff and rangers, who indicated more consistent sightings of Sclater's monals at this site compared with the southern part of the Gaoligong Mountains (Luo and Han 2011). The higher density was assessed through camera trap surveys, which reported increased Sclater's monal populations in this central area (Gao et al. 2017; Li et al. 2022). The terrain is complex, featuring numerous valleys and cliffs, with an average slope of about 40°. The average temperature of our study site from March to May was around 5.6°C (range: 0.1°C–25.9°C), and its annual precipitation could reach up to 3300 mm (Xue 1995). In late April 2016, the snow depth at the research site ranged from 1.5 to 42 cm. The vegetation mainly consists of bamboo forests, mixed with a small amount of Cangshan fir (*Abies delavayi*), cedar (*Sabina pingii*), as well as rhododendron bushes and ground cypress (*Sabina procumbens*), and other shrubs (Xue 1995).

### 2.2 Searching for Nests

Based on our previous observation, the breeding season of the Sclater's monal is from March to May (Luo and Han 2011). We conducted our fieldwork from March to June in 2015 and 2016. During the early breeding season (i.e., early March) in 2015, we identified the broad activity area of the Sclater's monal. We did this by monitoring calls and songs, detecting activity traces, and searching for suitable nesting habitats. We then narrowed our focus areas to a total area of approximately 5 km^2^ for a thorough search for nests (Figure 1). We used binoculars and telescopes to search for potential nesting sites in rocky cliffs, and other potential habitats, including fallen woods and rocky valleys (Luo and Han 2011). When an active nest was found, its breeding status was determined based on the parent bird's behavior, such as egg laying and incubating. If old eggshells were found inside the nest, we classified it as a reused nest (Luo and Han 2011). During the field search in the focused area, whenever possible, we also identified inactive reused nests. In these nests, we found old monal eggshells, but no parent monals used the nests in that year.

### 2.3 Nest Measurements

We measured the three active nests after all fledglings had departed. We first noted the elevation and the slope of each nest. Sclater's monal nests are typically found on small platforms while located on larger rocky cliffs (Figure 1). We measured the maximum length and width of these cliffs using a rangefinder (model: SW‐600A) or a tape measure (50 m). We also measured the height of the nesting platform above the ground with the tape. Finally, we used a caliper with an accuracy of 0.02 cm to measure the length and width of the elliptical‐shaped nest.

### 2.4 Incubation Behavior

After locating an active nest, we placed a camouflaged tent as the observation shelter 20 to 50 m away from the nest to observe the incubation behavior of the breeding pair while minimizing disturbance to their breeding activities. Due to personal constraints and the long incubation duration and intervals, incubation behavior can easily be missed by researchers. We thus relied on temperature data loggers (Datalog ZDR‐21, Zhejiang, China) with two probes to record the temperatures inside and outside the nests. This enabled us to determine the timing of off‐nest and on‐nest events of the incubating female. To do this, one probe was placed into the bottom of the nest to record the temperature inside, and another was suspended outside the nest to record the ambient temperatures. In 2015, we placed two temperature loggers in two nests during the early breeding stage. To minimize disturbance, we did this when the female birds were away from the nests and removed the logger after the completion of the breeding (personal observation). Sclater's monal tends to reuse its nesting sites, so we installed a temperature logger to the same two nests before egg laying in 2016. Only one of them was reused in 2016.

We collected incubation data from three out of four active nests, as we were unable to measure incubation behavior for another active nest in 2016 because the breeding was nearing completion by the time it was located. We recorded the entire incubation period (32 days) for Nest ID. 2_2016 and recorded 18 and 14 days for Nest ID. 1_2015 and Nest ID. 2_2015, respectively. We defined the female's departure from the nest as occurring when the temperature inside began to decrease continuously, while the ambient temperature did not show such trends. Conversely, we defined the female's return to the nest as occurring when the temperature inside continuously increased. We validated some of these timings through our observations of the incubating females' departures and returns. The average temperature change between females' departures and returns was 5.7°C. We then calculated the duration (in min) of each on‐bout and off‐bout for each nest and incubation day. For the three nests in which we only recorded parts of the incubation periods, we back‐calculated the incubation day for the emergence of each incubation on‐bout and off‐bout by assuming the whole incubation period was 32 days. Due to the small sample sizes, we compared the on‐bout and off‐bout duration's among nests using the nonparametric Kruskal–Wallis and pairwise Wilcox rank‐sum tests. We did our analyses using package “stats” in R and package “ggplot” for data visualization (R Core Team, 2020; Wickham 2016).

### 2.5 Other Breeding Behaviors

We encountered one intense male–male interaction in 2015. Throughout the breeding season in 2016, we observed the breeding behaviors of the incubating pairs, and their interactions with other monal individuals in their territory from the shelter during different stages of breeding, including preincubation, early incubation (Day 1–Day 16), late incubation (Day 17–Day 32), and postincubation. When we encountered rare behaviors, such as male–male fighting, and courtship displays, we recorded the duration of the behavior and noted the breeding stages of the nest. We also took pictures and made videos of the behaviors. In addition, we installed five camera traps (model Ltl‐6210) in the research areas to assist in recording the breeding behaviors of the monals.

## 3 Results

### 3.1 Nesting Site Use

We found three active nesting sites, including one (Nest ID. 1, Figure 1) only used in 2015, and the second one (Nest ID. 3, Figure 1) only found in 2016 but could be used in 2015 as well because we found old eggshells in 2016, and two hatchlings near this nest on May 22, 2015. Another nest was used in both years (Nest ID. 2_2015; Nest ID. 2_2016). In addition, we found two (Nest ID. 4 and 5) and one (Nest ID. 6) inactive nesting sites in 2015 and 2016, respectively. In these inactive nests, there were some old, cracked eggshells but no breeding monals used them during the study period.

The nests were built on big rocky cliffs, on a sunken south‐facing platform. The elevations of the nests ranged between 3535 and 3892 m (Figure 1). We measured the three large nesting rocky cliffs as follows: 15 m × 8 m (maximum length × maximum width), 15 m × 20 m, and 16 m × 21 m. The average height of the nesting platforms is 6.97 ± 1.49 m (*n* = 3) above the ground. The sizes of nests were 67.5 × 59.5 cm (length × width), 73.5 × 52.5 cm, and 75.8 × 31.95 cm, respectively (Table S1).

### 3.2 Eggs and Incubation Behaviors

**FIGURE 2:**
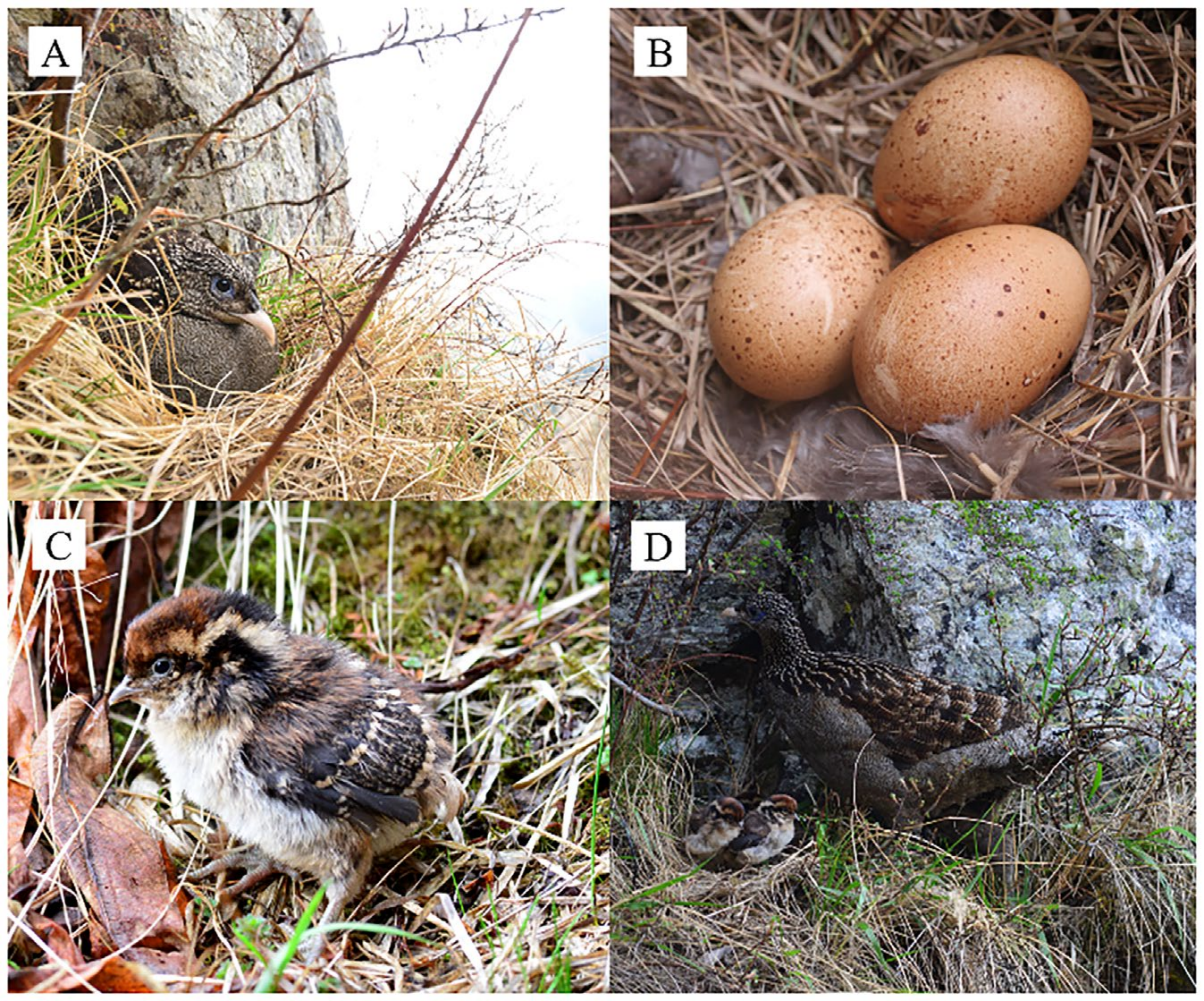
Eggs, nest, nestlings, the incubating female of the Sclater's Monal (A, incubating female, B, nest and eggs; C, 1‐day‐born nestling; D, nestling and female bird preparing for leaving the nest, the photograph was captured by Wang Bin).

The surface of the eggs was dark yellow, unevenly covered with brown spots of different sizes and shapes, which were incubated only by females (Figure 2A,B). The clutch size was 2.75 ± 0.5 (2 or 3 eggs; *n* = 4 nests at three nesting sites); the hatchability was 100% (*n* = 11 eggs); and the measured weight of eggs was 68.41 ± 6.47 g (*n* = 6 eggs from 2 nests). For the nest (Nest No. 2, 2016) that we monitored throughout the entire incubation period, the incubation period was 32 days, from April 30 to May 31, 2016. For the other three nests with known hatching dates only, we back‐calculated the start of the incubation. On average, the female monals initiated incubation from March 30 to April 30, and the hatchlings left the nests from May 1 to May 31 (Figure 2C,D).

**FIGURE 3:**
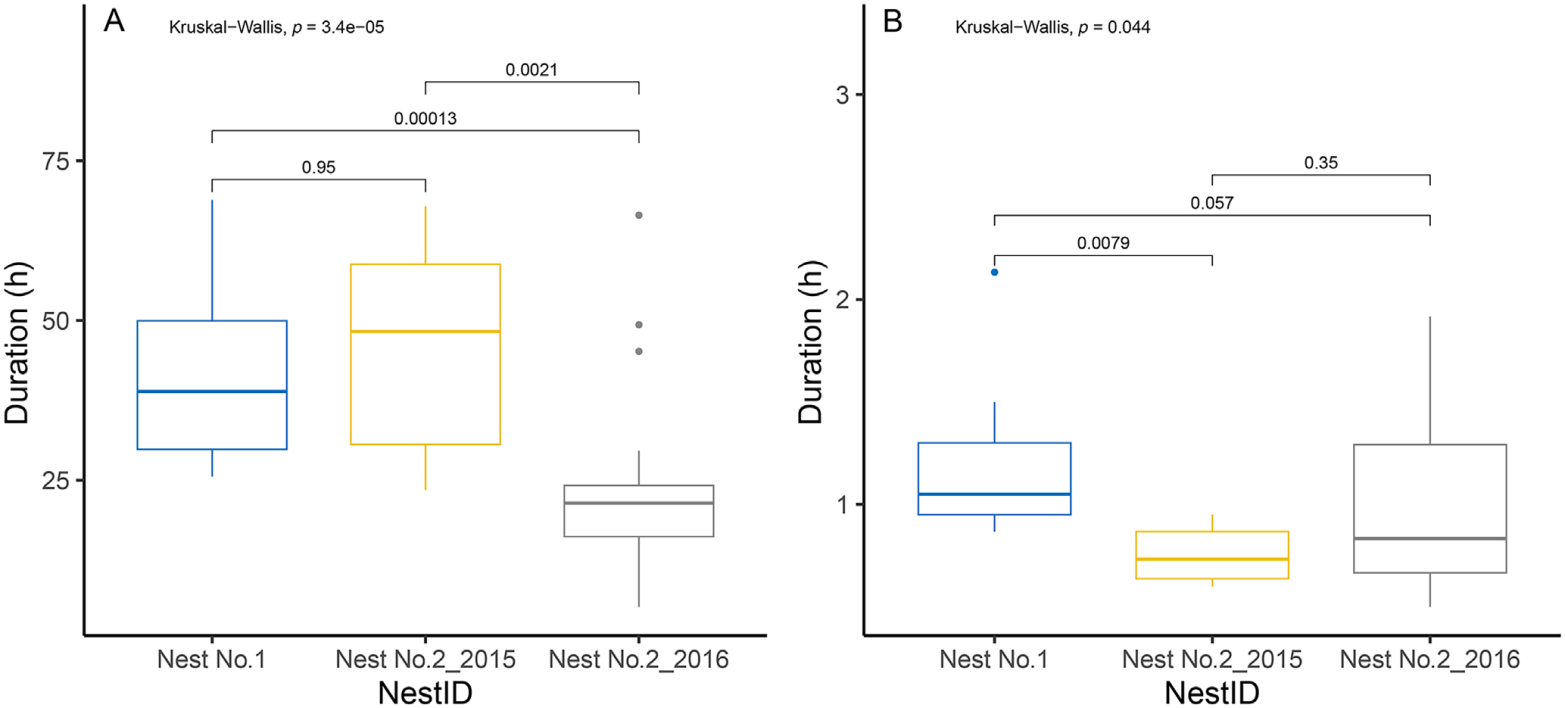
Durations of on‐bout and off‐bout in each nest of monals. (A: On‐bout duration; B: Off‐bout duration).

We monitored the incubation using an automatic temperature recorder in three active nests for a total of 64 days (18–32 days). We were not able to monitor another active nest as the nestlings were fledged right after we found it. The incubating female monal left the nest once a day or once after a day for foraging. Among the three nests, the female monal spent an average of 97.2% (range = 95.7% [Nest No. 3] – 98.4% [Nest No. 2_2015]) of the total time in incubation (i.e., on‐bout) in the nests, and 2.8% (range = 1.6% [Nest No. 2_2015] – 4.3% [Nest No. 3]) of the total time off the nest for foraging (off‐bout). The average duration of the on‐bout incubation was 25.69 ± 13.79 h (*n* = 43 on‐bouts), and the average time of the off‐bout was 1.04 ± 0.45 h (*n* = 46 off‐bouts). The duration of each on‐bout incubation varied among nests (Figure 3A). Specifically, the female monal of Nest No. 1 stayed on‐bout longer than the female monal of Nest No. 2, 2016. The duration of each off‐bout also varied among nests, and the female monal of Nest No. 1 stayed off‐bout longer than the female monal of Nest No. 2_2015 (Figure 3B).

### 3.3 Male–Male Interactions

During the breeding season in 2016, we documented 45 male–male interactions. Of these, 36 instances involved a male chasing another, presumably an intruding male, in flight. These chases, which were often followed by calls, could extend to 3 km in distance. The majority of these interactions were observed in April (25 instances), with fewer occurring in May (11 instances). We also recorded nine intense male–male fights, with one additional case in 2015. These involved two or three males engaging in physical combat within bushes, on rocks, or among bamboo forests. The fighting behavior included aerial engagements, where the birds rise 0.5–1 m above the ground and use their claws and beaks to attack one another. The average duration of these fights was 57 min (range: 5–137 min). The most intense fights were observed on days when incubating females were expected to lay eggs or had just begun incubation. For instance, on April 22, 2015, we observed a fight with 37 attacks between two males over 133 min on Day 2 of incubation for Nest ID. 2_2015. Similarly, on May 2, 2016, corresponding to Day 2 of incubation for Nest ID. 2_2016 and Day 19 for Nest ID.3, we recorded three separate fights throughout the day, a total of 52 attacks over 283 min.

### 3.4 Courtship Displays

We observed 16 courtship displays from April to June 2016. Of these, nine displays occurred between April 28 and May 2, coinciding with the start of incubation for one nest (Nest ID. 3). One display took place on April 3 before either of the two females in the two nests started indicating. Four displays happened between April 18 to 21, during which one female had laid eggs and the other had not yet started incubation. In addition, two other displays occurred after the completion of both nests, on June 2.

Each display often consisted of two phases. In the first phase, the male gradually approached the female from a distance of 20–5 m, then circled the female, moving back and forth while slowly nodding and pecking. This phase lasted an average of 877.08 ± 1061.54 s (range: 10 s to 59 min; *n* = 13). During this time, the male's facial wattles enlarged and became a sparkling blue. In the second phase, the male displayed in front of the female at a close range (approximately 1 m), by shrinking his neck, spreading tail feathers, and continuously flapping its wings up and down while swinging its raised tail feathers back and forth. This process was relatively brief, lasting about 10 s to 2 min. However, in all cases, the female did not respond to the displaying male, and no copulation was observed. The male then retracts his wings and tail feathers, turns away from the female, stands upright, droops his tail, lifts his feet, and hops away (Figure S1A–E).

In addition, in one instance, the male monal was displaying to the incubating female in the nest. Also, we recorded four displays where subadult male monals were also involved in four of these courtship displays.

## 4 Discussion

### 4.1 Nest Site Selection

For the successful breeding of Sclater's monal, the selection of an appropriate nesting site is essential (MacDonald et al. 2016; Maisey et al. 2016). This decision could also be influenced by several environmental factors, including temperature, predation, and the availability of nest sites (MacDonald et al. 2016; Gao et al. 2022). Female choice of nest site has a major effect on hatching success and population density in galliforms (Cong and Zheng 2008). Our observation suggests that certain features such as slopes, rocky cliffs, and nesting platform features may play vital roles in nest site selection.

Sclater monal carefully selects south‐facing slop for nesting sites to avoid harsh weather and freezing temperatures in alpine environments, benefitting from increased solar exposure (Luo and Han 2011). These warmer, sun‐exposed sites provide favorable conditions for breeding, similar to the habitat preference of the Himalayan snowcock ( *Tetraogallus himalayensis* ) (Fu, Dai, et al. 2017; Fu, Liu, et al. 2017; Yao et al. 2017). In our study, all six monal nests were located on south‐facing cliffs, helping maintain ideal temperatures for embryo development, and reducing thermoregulation challenges for hatchlings (Tulp, Schekkerman, and Leeuw 2012; de Zwaan et al. 2020). Additionally, these slopes tend to become snow‐free earlier in the season, offering earlier nesting opportunities (de Zwaan and Martin 2018). While the availability of these sites is important, choosing thermally favorable nesting sites probably helps with the growth and survival of the offspring (de Zwaan and Martin 2018).

Camera trap surveys in this region, have shown that ground‐dwelling carnivores, such as weasels ( *Mustela sibirica* ), yellow‐throated marten (Martes flavigula), and leopard cats ( *Prionailurus bengalensis* ), inhabit the same habitats as Sclater monal (Gao et al. 2017; Li et al. 2022). While nesting on cliffs offers some protection, it is not foolproof; weasels can access these nests posing a significant threat to the birds and their eggs (King and Powell 2006). Luo and Han (2011) reported that a Hen harrier ( *Circus cyaneus* ) attempted to attack a Sclater's monal nest but was unsuccessful due to the defensive behavior of the incubating female. Her aggressive posture deterred the predator, causing it to abandon the attack. This indicates that despite the vulnerability of cliff nests to predation by corvids and raptors (Martin 1993; Mallord et al. 2008), female monals try to protect their nests, eggs, and chicks (Luo and Han 2011). While cliff nesting does not eliminate predation risk, it may reduce risk and is likely an essential strategy for enhancing breeding.

### 4.2 Limited Nesting Site Availability

During our study period, we observed intense male–male flighting, one lasting more than an hour. This behavior may suggest limited nesting site availability, similar to territorial fighting between males of White‐tailed Ptarmigans ( *Lagopus leucura* ) (Wilson and Martin 2008). Another possible reason for male–male competition is to gain access to females, which has been noted in many species (Soley and Eberhard 2021). However, the female monals were not present when fightings were observed, and the sex ratio of Sclater's monal was reported to be 1:1.07 in the field (Lu, Liu, and He 1991). This suggested that males were competing for suitable nesting sites, than to gain female access, indicating that resource scarcity is a major factor in the social dynamics of the species.

Another evidence of the limited nesting sites availability was the reuse of nesting sites. Broken eggshells from previous years at three nests, indicating nest reuse was not uncommon, consistent with earlier investigations (Luo and Han 2011). While this behavior indicates that competition for nesting sites is significant, the sample size of three to six nests might not accurately represent the overall nesting site requirements of species. These findings highlight Sclater's monal's adaptability and site fidelity in the alpine environment. Understanding these dynamics will be crucial for conservation efforts and for evaluating the population status of this regionally threatened species.

### 4.3 High Incubation Attendance

Incubation is an essential part of the life history of birds and requires a significant energy investment (Wiebe and Martin 2000; Jin et al. 2024). Species‐specific investments in incubation strategies, such as nest attendance, can differ based on environmental factors and individual traits (Amininasab et al. 2016; Gao et al. 2022). The average duration of the on‐bout incubation was longer than a day (25.69 ± 13.79 h). This extremely long nest attendance of Sclater Monal (97.2%) was found in our study and that of Luo and Han (2011). This is considered to be longer than any other alpine breeding pheasants, such as the Sichuan partridge, Blood pheasant, and Snow partridge (< 85% of nest attendance) (Jia, Liu, and Zhang 2010; Jia, Sun, and Swenson 2010; Fu, Dai, et al. 2017; Fu, Liu, et al. 2017; Li et al. 2022), and lowland breeding pheasant such as the Reeves's pheasant ( *Syrmaticus reevesi* ) (93.11% of nest attendance) (Jin et al. 2024). In some galliforms, high nest attendance throughout incubation guarantees steady and quick development of the embryo (Jin et al. 2024), while also posing less exposure to potential predators (Fontaine and Martin 2006). The fact that they only spent an average of 1 h off the nest for foraging was probably because their embryo had not developed a good tolerance to low temperatures as what Sichuan Partridge and Blood pheasant did (Jia, Liu, and Zhang 2010; Jia, Sun, and Swenson 2010; Fu, Dai, et al. 2017; Fu, Liu, et al. 2017). This low frequency of recesses and a high degree of nest attendance could potentially mitigate predation pressure, decrease the frequency of egg warming, and limit the physiological expenses incurred by female monal. However, due to limited comparative data for similar alpine species, we recognize that further research is necessary to determine whether the longer incubation period in Sclater's Monal is related to its high nest attendance or if it reflects other adaptive strategies.

## 5 Conclusion

In conclusion, our research offers valuable insight into breeding ecology and life‐history strategies of the Sclater's monal in the alpine environments of the Gaoligong Mountains in Southwest China. The selection of south‐facing slopes for nesting sites highlights the adaption of Scalater monal to harsh weather, improves embryo development, and reduces predation risks. High incubation attendance by female monals indicates they have increased parental care than other pheasants. Furthermore, the fierce competition between males and the reuse of nesting sites suggests that suitable nesting sites are limited, which may affect population dynamics. All of these insights are crucial for conserving this regionally threatened pheasant.

## Author Contributions

**Nehafta Bibi:** formal analysis (supporting), writing – original draft (lead), writing – review and editing (lead). **Ge Gao:** data curation (lead), methodology (lead). **Dan Liang:** formal analysis (lead), investigation (lead), methodology (supporting), writing – review and editing (supporting). **Xu Luo:** conceptualization (lead), funding acquisition (lead), project administration (lead), resources (lead), supervision (lead), writing – review and editing (equal).

## Ethics Statement

The study was carried out under the Institutional Animal Care and Use Committee at Southwest Forestry University. No nests or nestlings were harmed during our fieldwork.

## Conflicts of Interest

The authors declare no conflicts of interest.

## Supporting information


Data S1

## Data Availability

Necessary data for this study are available as Supporting Information.
