# Supplementary material for: High Incubation Attendance and Nesting Site Constraints of the Sclater's Monal in an Alpine Environment in Southwestern China
Source: Ecol Evol. 2024 Dec 4;14(12):e70665. doi: 10.1002/ece3.70665 (PMC11617325; doi:10.1002/ece3.70665)
Supplement: Supplementary file 1 — Data S1 [file ECE3-14-e70665-s001.docx]

Table S1. Nesting site characteristics of the Sclater’s Monal

| Nest site ID | Altitude (m) | Rock face | Rock size (m) | Nest height (m) | Nest size (cm) | Active year | Clutch size | Confirm Information |
| --- | --- | --- | --- | --- | --- | --- | --- | --- |
| 1 | 3535 | southwest | 15 ×8 | 5.29 | 67.5 ×59.5 | 2015 | 3 | Active in 2015 |
| 2 | 3792 | southwest | 15 ×20 | 7.5 | 73.5 ×52.5 | 2015/2016 | 3/2 | Old eggshell found in 2015 |
| 3 | 3823 | southeast | 16 ×21 | 8.12 | 75.8 ×31.95 | 2016 | 3 | Old eggshell found in 2016 |
| 4 | 3892 | southwest | 15 ×35 |  | - | Before 2015 | -- | Inactive in 2015-2016, but old eggshell found in 2015 |
| 5 | 3802 | west | 25 ×30 |  | - | Before 2015 | -- | Inactive in 2015-2016, usage confirmed by field guide |
| 6 | 3700 | southwest | 20 ×25 |  | - | Before 2015 | -- | Inactive in 2015-2016, but old eggshell found in 2015 |

*Rock size: Width × height; Nest size: Length × width


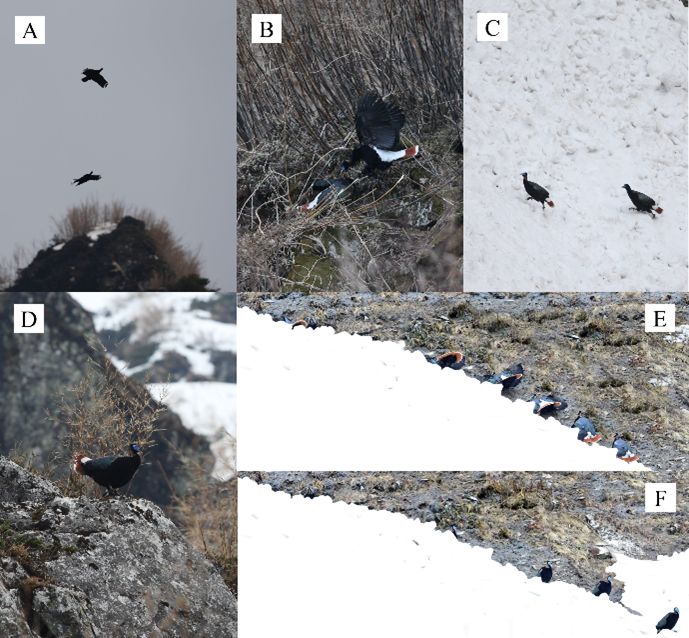


Fig S1. Male-male fight and courtship display of Sclater’s Monal (A, fighting in flight; B and C, fighting on the ground; D and E, the display of adult male; F, the male jumping off after a courtship failure. E and F were a series of photo shoots showing the continuity of display behavior and its subsequent behavior after failure. Photography by Xu Yong and Wang Bin)
